# Report of Cases

**Published:** 1847-01

**Authors:** Frederick Hyde

**Affiliations:** Cortlandville, N. Y.


### ART. III.—*Report of Cases, by* Frederick Hyde, M. D., *of Cortlandville, N. Y.*

Dr. Flint:—I send you the following cases, although somewhat minor in their character.

J. H. B. of Scott, Cortland Co., æt. 14, and healthy, received on the 25th of Feb., 1846, a transverse wound, made with a pocket knife, about an inch and half in length, near and just above the wrist; the wound being deepest upon the ulnar side of the arm. But little blood was lost at the time the wound was made, and that was venous. Wound was closed and the ordinary dressings applied; union took place and the parts appeared sound. In a few days the patient returned to his surgeon (being just three weeks from the infliction of the wound,) with a pulsating tumor, about the size of a large pea, in the track of the wound directly over ulnar artery. Patient said it had only been there a day or two. By March 30, it had attained the size of a hickory nut. Gentle pressure was applied to the tumor, the fourth day from which sloughing occurred, with profuse bleeding from the ulnar artery; it was arrested by pressure, but in forty hours the bleeding returned. It was again restrained by pressure until I saw him on 5th April, in consultation with Drs. Bradford and Maxon, who were in attendance.

Having ascertained that pressure upon the ulnar artery immediately above the tumor would not control the blood, also that the brachial artery had not a high bifurcation, and that pressure upon the same at the middle of the arm stopped at once the bleeding, and all pulsation in both the ulnar and radial arteries, our conclusion was to tie the brachial artery. At the middle of the arm an incision two and a half inches in length was made, corresponding to the inner edge of the biceps muscle; the artery readily exposed and tied, when all pulsation at the wrist ceased. Wound was dressed, arm placed in comfortable position, at 6, P. M. Attending surgeon said that in six hours from the operation the hand grew warm, that in twelve hours the radial artery pulsated feebly at the wrist.

April 8th, 8, A. M. Radial artery pulsated distinctly, no pulsation in the tumor and reduced three fourths in size. At 9, A. M. bleeding returned slightly, was readily arrested by gentle pressing. At 3 P. M. I saw him again, found the tumor two-thirds its size previous to the operation; while examining the tumor the blood again flowed moderately. Compression upon the ulnar artery directly above the tumor did not stop the bleeding, but when applied to the vessel below the sac upon sound parts, just as the artery passed the lower margin of the annular ligament, it stopped the pulsation and controlled the blood entirely, and when applied to the radial artery the result was the same. It was evident that the blood came from the distal extremity of the artery; a compress was therefore put directly over the vessel as it passed the annular ligament, where the parts were sound, which controlled effectually the blood. Aneurismal sac suppurated; the parts became healthy; wound in the arm united mainly by first intention, ligature came off the twentieth day. From this time the case progressed favorably, and he has now a perfect use of his hand and arm.

The facts in this case show no more clearly the ability of the collateral branches to carry on the circulation, when its progress in the main artery is entirely obstructed, than that they may re-establish the circulation so soon as to produce a return of the hemorrhage, before the adhesion in the opened artery is perfected. Why did not the ulnar artery bleed from its proximal extremity? We find the answer to this question in the Anatomy of the Arteries. When we see that the profunda major is the main source of supply for the recurrent circulation upon the radial side, while the opposite side depends upon the profunda minor and anastomatica, through the ulnar recurrent for its blood, we have the reason why the radial circulation was so much easier than the ulnar; so, too, when we come down to the hand, and see the ample provision there, in

Page(s) missing the free anastamosing of its vessels—the query is at once solved why the bleeding in this case returned upon the distal side of the tumor, and not upon the proximal.

Case 2d. A. M., æt 18; in good health; resides in Groton, Tompkins Co. On the 13th May, 1846, he received a wound in the top of the foot, from an ax. It bled profusely; faintness ensued, which, together with pressure, controlled the bleeding. 14th, at 9, P. M., bled again—it was stopped as before. 16th, at 7, A. M., bleeding returned, and was again controlled by pressure. Dr. J. Goodyear now saw the patient for the first time. In two hours it bled again. Pressure and elevation of limb controlled the blood as before. I saw him at 5, P. M. of the same day. Patient pallid; had evidently lost much arterial blood. The dressings were removed from the wound, which was two inches in length, on a line with the interspace between the first and second metatarsal bones. Wound was gaping, and filled with a mass of coagula, projecting above the surface, and pulsating strongly. The surrounding parts were inflamed; pressure upon the anterior tibial artery did not lessen the pulsation in the wound, but when applied to the posterior tibial, it immediately ceased; did not bleed during the night. 17th, at 8, A. M. The mass of coagula in wound considerably augmented, and pulsating more strongly. In consultation with Drs. Mac and J. Goodyear, although there had been no bleeding for a little more than eighteen hours, the conclusion was that any treatment would be unsafe, short of ligature to the posterior tibial artery. An incision was made two inches in length behind the internal malleolus; the artery exposed, and the ligature applied. All pulsation in the wound at once ceased,—from this the case progressed well; ligature came off the seventeenth day, and the use of the leg and foot is complete. Why did not pressure control the bleeding? We will answer—the point of opening from which the blood escaped was situated unfavorably for the direct application of pressure. The cutting instrument passed down between the bones, and the vessels opened was evidently the internal plantar artery. It is very likely, too, that the vessel was not divided across, but an opening made through one of its walls. This would also increase the chances for persistent hemorrhage. At the time

I saw him, the whole wound was decidedly unhealthy; conflicting directly with any sort of procedure to arrest permanently the bleeding, short of intercepting the blood before it reached the unhealthy wound.

*Cortlandville, Dec.* 12, 1846.

